# Surgery and Resource Utilization Trends for Pediatric Intussusception From 2005 Through 2014

**DOI:** 10.7759/cureus.10611

**Published:** 2020-09-23

**Authors:** Parth Bhatt, Priyank J Yagnik, Pavithra Saikumar, Narendrasinh Parmar, Mihir Dave, Jason K Amponsah, Neel S Bhatt, Mayank Sharma, Badal Thakkar, Keyur Donda, Fredrick Dapaah-Siakwan

**Affiliations:** 1 Pediatrics, United Hospital Center, Bridgeport, USA; 2 Pediatrics, University of Kansas School of Medicine-Wichita, Wichita, USA; 3 Pediatrics, Brookdale University Hospital and Medical Center, Brooklyn, USA; 4 Internal Medicine, University of Nevada Reno, School of Medicine, Reno, USA; 5 Public Health, Emory University School of Medicine, Atlanta, USA; 6 Pediatric Hematology and Oncology, University of Washington School of Medicine, Seattle, USA; 7 Pediatrics, University of Miami, Miami, USA; 8 Internal Medicine, Sinai Hospital of Baltimore, Baltimore, USA; 9 Pediatric, University of South Florida, Tampa, USA; 10 Medicine/Neonatology, Valley Children’s Healthcare, Madera, USA

**Keywords:** intussusception, trends, incidence, surgery, enema

## Abstract

Background: Air or barium enema reduction is becoming increasingly common and safer for pediatric intussusception. However, little is known about trends of pediatric intussusception requiring surgical intervention in the United States.

Methods: National Inpatient Sample database was analyzed from 2005-2014 to identify pediatric (≤18 years) intussusceptions along with procedures such as enema and/or surgical intervention. Trends in the rates of surgical intervention were examined according to encounter-level (age, gender, race, comorbidities) and hospital-level (hospital census region, teaching status) characteristics. Outcomes of pediatric intussusception requiring surgical intervention were analyzed in terms of length of stay and cost of hospitalization. Factors associated with surgical intervention were also analyzed. P value of < 0.05 was considered significant.

Results: Out of 21,835 intussusception hospitalizations requiring enema or surgical intervention, 14,415 (66%) had surgical intervention; 90% of which (12,978) had no preceding enema. Surgical intervention rates among intussusception hospitalizations varied by age (highest < 1 year), gender (male > females) and race (Hispanics > Whites and Blacks). During the study period, overall surgical intervention rate remained stable (2.2 to 1.7, P=0.07) although it declined in those under 1 year of age. Children with severe disease, gastrointestinal comorbidities over the age of 4 years had increased odds of surgical intervention, whereas hospitalization in large and urban teaching hospitals had decreased odds of surgical intervention. Length of stay and hospital cost remained stable from 2005-2014.

Conclusion: The rates of surgical intervention and resource utilization for pediatric intussusception remained stable from 2005-2014, however they declined significantly in infants. The proportion of intussusception hospitalization requiring surgery remains high and further studies are needed to explore the possible factors.

## Introduction

Intussusception, defined as invagination of one intestinal segment into another, is the second most common cause of intestinal obstruction in children. If untreated, intussusception can lead to ischemia, perforation, and peritonitis with systemic complications [1]. The incidence of intussusception is estimated to be 74 per 100,000 children under 1 year of age in the United States (US) with a range of 9 - 328 per 100,000 children depending on the data source and the country where the study was performed [1].

Definitive management of intussusception is accomplished by either reduction with the administration of enema (pneumatic or hydrostatic with saline or contrast) under fluoroscopic guidance or surgical treatment [2]. Surgical intervention (SI) is reserved for cases with pathological lead points, intestinal perforation, unsuccessful reduction or hemodynamic instability [2]. The surgical intervention rate for intussusception in the United States declined by 38% from 26 to 16 per 100,000 infants between 1993 and 2004 [3]. A recent meta-analysis showed most studies on intussusception did not include surgical intervention as a study question and there is the need for research on surgical management of pediatric intussusception. In recent years, several studies have reported the practice of same-day discharge from the emergency room (ER) or short stay units after successful reduction of intussusception to be safe [4-5]. There is a paucity of data on the extent to which outpatient management of intussusception after successful reduction impacts the trends in hospitalization rates and surgical interventions for intussusception in the US.

We therefore aimed to study the epidemiological, inpatient management, and resource utilization trends in hospitalized children with intussusception in the US from 2005-2014 using the National Inpatient Sample (NIS).

## Materials and methods

Study design and data source

We performed a retrospective serial cross-sectional study using the NIS database from 2005-2014. The NIS database is part of the Healthcare Cost and Utilization Project (HCUP), sponsored by the Agency for Healthcare Research and Quality (AHRQ). In 2014, the NIS sampling frame consisted of 44 states and the District of Columbia covering more than 96% of the US population [6]. The unit of analysis in the NIS is inpatient stays, not individual patients. Each individual hospitalization in this database is de-identified and maintained as a unique entry with one primary discharge diagnosis, <24 secondary diagnoses, and <15 procedural codes during that hospitalization. The NIS database has been used to study hospitalization trends in neonatal, pediatric, and adult populations [7]. This study involved subjects from a de-identified database, so it was exempted from Institutional Review Board (IRB) review.

Study population

**Figure 1:**
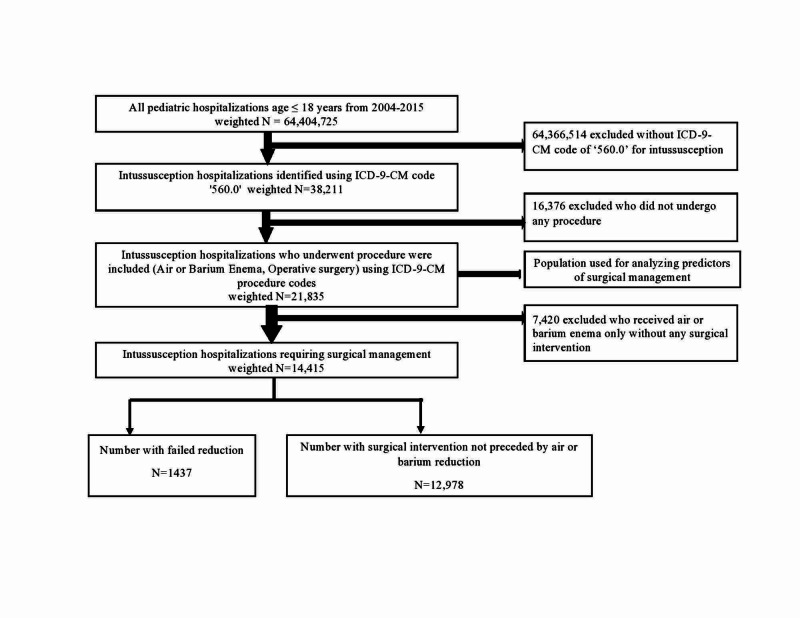
Population derivation ICD-9-CM- International Classification of Diseases, Ninth Revision, Clinical Modification

Our cohort of pediatric hospitalizations (≤18 years) was derived from the years 2005-2014 of the NIS database which has been represented in two-year epochs. Further, to limit double-counting, transfers to short term hospital, skilled nursing facility, intermediate care facility, and another type of facility were excluded using 'DISPUNIFORM' variable. In keeping with previous studies, intussusception hospitalizations were identified using the International Classification of Diseases ninth revision, clinical modification (ICD-9-CM) diagnosis code '560.0' in any of the diagnosis fields [5,8]. Previous studies have demonstrated this code to be sensitive and highly specific in addition to having a high positive predictive value [9]. ICD-9-CM procedure codes were used to identify non-surgical interventions such as air/barium enema (96.29 and 87.64) and surgical interventions (45.00-45.03, 45.60-46.30 and 46.80-46.82) in intussusception hospitalizations. A similar methodology has been used in the past [10]. Hospitalizations which had both non-surgical and surgical interventions were labeled as failed air/barium enema requiring surgery. Figure 1 depicts population derivation flowchart.

Definition of variables

We studied patient encounter level and hospital level characteristics of the study population. Patient encounter level characteristics included age, gender, race, median household income, and primary payer status. Data was further stratified based on age (<1 yr, 1-2 yrs, 2-4 yrs, >4 yrs) and race (White, Black, Hispanic, and others which included Asian, Pacific Islanders, and Native Americans). Hospital-level characteristics included hospital region, hospital location, teaching status, and hospital bed size. The NIS database stratifies hospital admission by US census regions of Northeast, Midwest, South, and West as practice patterns vary by census regions. The NIS collects information regarding point of origin as well as final disposition of a hospitalization. This information helped to differentiate between hospitalizations that were ‘transferred out to outside facility’ and those which were ‘transferred into the hospital from outside facility’. Non-newborn hospitalizations that were transferred into the hospital were defined using the admission source ‘ASOURCE’ or point of origin ‘PointOfOriginUB04’ and ‘TRAN_IN’ variable based on data availability [7]. Hospitalizations with bowel ischemia/gangrene, bowel perforation, peritonitis, and sepsis were labeled as severe disease. Gastrointestinal (GI) comorbidities were identified as per previous literature [10]. Complex chronic conditions (CCC) have been previously shown to be confounder and were identified using relevant ICD-9-CM codes [11].The NIS provides charges, which is the amount the hospital billed for hospitalization, and it does not reflect the actual costs, i.e. expenses incurred in providing hospital services, such as wages, supplies, and utility costs. To analyze costs, we utilized Cost-to-Charge Ratio (CCR) files provided by the NIS and multiplied charges with CCR [12]. Cost for each year was calculated in terms of 2014 cost after adjusting for inflation according to consumer price index data released by the US government [7].

Statistical analysis

Baseline characteristics of intussusception hospitalizations were compared according to surgical management during admission. Continuous variables were compared using medians and interquartile range (IQR) and categorical variables were compared using proportions. Chi-square test, and Student’s test or Wilcoxon rank sum test were used to estimate differences between categorical and continuous variables, respectively, as appropriate based on data distribution. Surgical intervention rate was calculated by dividing the number of intussusception hospitalizations requiring surgery by the corresponding subgroup population derived from the Center for Disease Control and Prevention’s Wide-ranging Online Data for Epidemiologic Research [13]. Differences in surgical rates for intussusception amongst the various subgroups were assessed using Analysis of Variance (ANOVA) with post-hoc Tukey’s Highly Significant Difference (HSD) for pairwise comparisons and Student’s t-test as appropriate. Survey procedures were used to account for the complex survey design of the NIS. Discharge weights (‘DISCWT’ variable) provided by NIS was used to generate national estimates of the intussusception hospitalizations for each study period. Due to a change in sampling and weighting strategies from 2012, HCUP has provided trend weights for the years 1993-2011 to make estimates comparable to the new design (2012 and after). For trend analysis, the Cochran Armitage test for dichotomous dependent variables and Jonckheere-Terpstra test for continuous dependent variables were used. Survey logistic regression was used to analyze predictors of surgical interventions. Hierarchical mixed effects logistic regression models were used to analyze the predictors of length of stay (LOS) and hospitalization cost for surgical management. Hierarchical regression is designed to deal with clustered or grouped data in which analytic units are naturally nested or grouped within other units of interest and helps to account for “within-cluster” correlations at each level of the hierarchy and properly adjust estimates to account for them. Statistical analysis system (SAS®) 9.4 (SAS Institute Inc., Cary, North Carolina) was utilized and P-value of <0.05 was considered significant for all analyses.

## Results

A total of 21,835 hospitalizations had a primary diagnosis of intussusception who had an enema and/or surgery from 2005 to 2014. Of these, 14,415 (66.0%) had surgical interventions. The baseline characteristics of all the intussusception hospitalizations requiring intervention are outlined in Table 1. In summary, 64.1% were males, 40.9% were of white race, 45.9% were less than 1 year of age, 54.7% had Medicaid, 11.2% had severe disease, 16.3% had GI comorbidities and 5.1% had CCC. The majority of children requiring surgery were admitted in large (65.2%), urban teaching (82%) hospitals in the South census region (37.1%). Hospitalizations transferred from one institution to another were more likely to require surgical intervention than nonsurgical intervention (19.5% vs 13.2%; P<0.001).

**Table 1:** Baseline characteristics of Intussusception hospitalizations in the United States, 2005-2014 IQR- Interquartile range, GI- gastrointestinal

	No-Surgery	Surgery	Total	p-value
Intussusception N (Unweighted)	4508	2982	7490	
Intussusception N (Weighted)	21910	14415	36325	
Patient related characteristics				
Gender (%)				0.8
Male	63.9	64.4	64.1	
Female	34.6	34.9	34.7	
Missing	1.4	0.7	1.2	
Race (%)				<.0001
White	38.1	40.9	39.2	
Black	11.9	9.9	11.1	
Hispanic	23.4	21.3	22.6	
Others	10.0	8.4	9.4	
Missing	16.7	19.4	17.8	
Age Median (IQR)	0.5 (0-1.6)	0.3 (0-3.0)	0.4 (0-2)	0.3
Age (Years)				<.0001
<1 year	30.2	45.9	36.4	
1- 2 year	18.9	14.4	17.1	
2-4 year	33.4	18.0	27.3	
>4 year	17.6	21.8	19.2	
Median household income category for patient’s zip code (%)				<.0001
1. 0–25th percentile	25.9	29.2	27.2	
2. 26–50th percentile	22.0	23.7	22.7	
3. 51–75th percentile	24.8	23.5	24.3	
4. 76–100th percentile	24.5	21.5	23.3	
Missing	2.8	2.2	2.6	
Primary payer (%)				0.002
Private	46.7	45.3	46.1	
Medicaid/Self-pay/Others	53.1	54.7	53.7	
Missing	0.2	0.1	0.2	
Co-morbidities				
Severe disease	0.9	11.2	5.0	<.0001
Bowel ischemia/gangrene	0.2	7.9	3.2	<.0001
Bowel perforation	0.0	3.0	1.2	<.0001
Peritonitis	0.0	0.6	0.3	<.0001
Sepsis	0.7	2.4	1.4	<.0001
Co-morbid GI conditions	5.2	16.3	9.6	<.0001
Chronic medical conditions of childhood	4.8	5.1	5.0	<.0001
Hospital related characteristics				
Hospital region				<.0001
North East	27.0	18.2	23.5	
Mid West	16.3	19.8	17.7	
South	30.6	37.1	33.2	
West	26.1	24.9	25.7	
Hospital bed size (%)				<.0001
Small	10.6	10.5	10.6	
Medium	23.4	23.3	23.4	
Large	65.6	65.2	65.4	
Missing	0.4	1.0	0.7	
Hospital location & Teaching status (%)				<.0001
Non-Teaching (Urban and rural)	13.6	17.0	14.9	
Urban - Teaching	86.0	82.0	84.4	
Missing	0.4	1.0	0.7	
Transfer from outside facility (Acute care hospital/Facility) (%)	13.2	19.5	15.7	<.0001

Table 2 categorizes the cases of intussusception according to their management modalities such as air/barium enema, failed air/barium enema requiring surgery, or surgical management alone. There were 7,419 intussusception hospitalizations managed with air/barium enema, out of which 1,437 (19.4%) subsequently had surgery due to failed reduction. Of all the hospitalizations that had an intervention, 59.4% (12,978/21,835) were managed with surgery alone. Table 2 also shows data on LOS, hospitalization cost, complications, and mortality with each treatment modality. The median LOS and hospitalization cost was the highest among surgically managed hospitalizations at 3.1 days and US $9,169, respectively, compared to those who underwent air/barium enema at 0.8 days and US $3,126 (P<0.0001), respectively. The highest complication rates were found among hospitalizations with failed enema requiring surgery and surgical management alone at 8.4% and 9.1% respectively, which was significantly higher compared to those who underwent enema at 1.5% (P<0.0001). In-hospital mortality was the highest among failed enema requiring surgery followed by surgical management alone at 1.1% and 0.6%, respectively (P=0.717). No mortality was noted among the enema group.

**Table 2:** Outcomes of different treatment modalities for Intussusception hospitalizations in United States, 2005-2014 aBonferroni post hoc test result for length of stay by treatment modalities of intussusception, Enema requiring surgery vs enema, P <0.0001; Enema requiring surgery vs surgery, P= 0.06 bBonferroni post hoc test result for cost of hospitalization by treatment modalities of intussusception, Enema requiring surgery vs enema, P <0.001; Enema requiring surgery vs surgery, P= 0.08 cBonferroni post hoc test result for post procedure complications by treatment modalities of intussusception, Enema requiring surgery vs enema, P <0.0001; Enema requiring surgery vs surgery, P= 0.7017 dBonferroni post hoc test result for mortality by treatment modalities of intussusception, Enema requiring surgery vs surgery P 0.7408; Enema excluded as no mortality noted. **Bonferroni correction analyzed using Proc GLM ANOVA procedure LOS- Length of stay, ANOVA- Analysis of variance

	Air or Barium enema	Failed Air or Barium enema requiring surgery	Surgical management (alone)	Surgical management
Unweighted (n)	1511	296	2686	2982
Weighted (N)	7419	1437	12978	14415
LOS^a^	0.8 (0.4-1.8)	2.7 (1.6-4.1)	3.1 (1.7-5.0)	3.0 (1.7-4.9)
Cost of hospitalization^b^	3126 (2127-4974)	9079 (6490-1287)	9169 (6203-14298)	9166 (6254-13975)
Complication after procedure (%)^c^	1.5	8.4	9.1	9.0
Mortality (%)^d^	0	1.1	0.6	0.6

Table 3 shows the trends in the incidence rate of surgical management of intussusception in terms of age groups, gender, race, and census region as well as resource utilization (LOS and hospitalization cost) from 2005-2014. The incidence rate of surgery among intussusception hospitalizations (expressed as per 100,000 of the subgroup population) varied significantly by age group (highest in <1-year group, P<0.001), sex (2.3 in males vs. 1.3 in females, P<0.001), and race (1.8 in Hispanics vs. 1.0 and 1.1 in whites and blacks, respectively, P<0.001). There was no variation among the census regions. Overall, there was no significant change in the rate of surgery for intussusception during the study period (2.2 to 1.7, P=0.07) and this remained unchanged after multivariable logistic regression analysis (adjusted odds ratio = 1.0, 95% confidence interval, 0.95-1.04, P=0.9) (Table 4). There was a statistically significant decline in the rate of surgery in hospitalizations of patients less than 1-year-old (20.3 in 2005-06 to 14.6 in 2013-14, P=0.01).

**Table 3:** Trends in incidence rate and resource utilization for surgical management of intussusception hospitalizations in the US from 2005 to 2014 ^ψ^P-value for ANOVA or Student’s t-test as appropriate ^Ω^All post-hoc pairwise comparisons with Tukey’s HSD significant with P<0.05 except for 1-2 years vs 2-4 years ^β^All post-hoc pairwise comparisons with Tukey’s HSD significant with P = 0.001 except for comparison between Whites and Black, P = 0.55 IQR- Interquartile range

	2005-06	2007-08	2009-10	2011-12	2013-14	Total	P-value ^ψ^	P-value for trend
Overall								
Overall	2.2 ± 0.2	1.8 ± 0.2	1.9 ± 0.2	1.7 ± 0.2	1.7 ± 0.1	1.9 ± 0.1		0.07
Age groups^Ω^							<0.001	
< 1 years	20.3 ± 3.0	16.2 ± 1.9	16.1 ± 2.0	15.3 ± 1.7	14.6 ± 1.0	16.5 ± 0.9		0.01
1-2 years	3.0 ± 0.4	2.6 ± 0.3	3.0 ± 0.4	2.1 ± 0.3	2.4 ± 0.3	2.6 ± 0.2		0.21
2-4 years	3.8 ± 0.6	2.9 ± 0.4	3.3 ± 0.5	2.9 ± 0.4	3.2 ± 0.3	3.2 ± 0.2		0.45
> 4 years	0.6 ± 0.1	0.5 ± 0.1	0.5 ± 0.1	0.5 ± 0.1	0.6 ± 0.1	0.5 ± 0.1		1.00
Gender								
Male	2.6 ± 0.3	2.2 ± 0.2	2.5 ± 0.3	2.1 ± 0.2	2.2 ± 0.1	2.3 ± 0.1	<0.001	0.21
Female	1.7 ± 0.2	1.3 ± 0.1	1.2 ± 0.2	1.2 ± 0.1	1.2 ± 0.1	1.3 ± 0.1		0.05
Race^β^							<0.001	
White	1.0 ± 0.2	0.8 ± 0.1	1.1 ± 0.2	0.9 ± 0.1	1.1 ± 0.1	1.0 ± 0.1		0.45
Black	1.1 ± 0.3	1.1 ± 0.2	1.1 ± 0.2	1.3 ± 0.2	1.0 ± 0.1	1.1 ± 0.1		0.78
Hispanic	1.9 ± 0.4	1.6 ± 0.2	2.2 ± 0.3	1.6 ± 0.2	1.5 ± 0.1	1.8 ± 0.1		0.21
Others	0.2 ± 0.04	0.2 ± 0.03	0.2 ± 0.04	0.2 ± 0.03	0.2 ± 0.03	0.2 ± 0.03		1.00
Hospital region							0.44	
Northeast	2.4 ± 0.6	2.0 ± 0.4	2.3 ± 0.7	1.6 ± 0.3	1.7 ± 0.3	2.0 ± 0.2		0.14
Midwest	1.7 ± 0.4	1.5 ± 0.3	1.6 ± 0.4	1.8 ± 0.4	1.8 ± 0.2	1.7 ± 0.2		0.21
South	2.5 ± 0.6	1.9 ± 0.3	1.5 ± 0.3	1.7 ± 0.3	1.6 ± 0.2	1.8 ± 0.2		0.14
West	2.1 ± 0.5	1.6 ± 0.3	2.3 ± 0.6	1.6 ± 0.3	2.0 ± 0.2	1.9 ± 0.2		0.80
Length of stay in days; Median (IQR)	3.2 (1.9-4.9)	2.9 (1.6-4.8)	2.9 (1.5-4.7)	2.8 (1.6-4.7)	3.1 (1.6-5.2)	3.0 (1.6-4.8)		0.45
Cost of Hospitalization in $; Median (IQR)	8909 (5867-13839)	8296 (5891-12077)	8840 (6037-12919)	10201 (7101-15228)	10350 (7217-17087)	9166 (6254-13975)		<.0001

 

**Table 4:** Multivariable logistic regression analysis with odds ratios and confidence intervals for factors associated with surgical intervention in pediatric hospitalizations with intussusception in the US.

Variable	Odds ratio (OR)	95% Confidence Interval	
		Lower limit	Upper limit
Year	0.96	0.94	0.99
Age in years			
<1 year	Reference		
1- 2 year	0.53	0.45	0.63
2-4 year	0.39	0.33	0.46
>4 year	0.69	0.58	0.82
Gender			
Male	Reference		
Female	0.96	0.86	1.07
Severe disease	11.18	7.84	15.93
Gastrointestinal Co-morbidities	3.77	3.10	4.59
Chronic medical conditions of childhood	0.75	0.56	1.02
Transfer from outside facility (Acute care hospital/Facility)			
No	Reference		
Yes	1.33	1.13	1.56
Primary payer (%)			
Private	Reference		
Medicaid/Self-pay/Others	1.00	0.89	1.56
Hospital Region			
Northeast	Reference		
Midwest	1.53	1.15	2.02
South	1.55	1.25	1.91
West	1.39	1.09	1.77
Hospital bed size (%)			
Small	Reference		
Medium	0.95	0.68	1.32
Large	1.04	0.78	1.39
Hospital location & Teaching status (%)			
Non-Teaching (Urban and rural)	Reference		
Urban - Teaching	0.78	0.62	0.98

Table 4 shows the factors associated with surgical intervention in intussusception hospitalizations. Age above 4 years, severe disease, GI comorbidities, and hospitalization outside of the Northeastern region had increased odds of surgical intervention for intussusception. However, age between 1 and 4 years, hospitalization in medium and large hospitals and urban teaching hospitals had reduced odds of surgical intervention.

Table 5 presents data on predictors of LOS and inflation-adjusted hospitalization cost in surgical cases of intussusception. Age >4 years, severe disease, CCC, complications from surgery and transfer from an outside facility were associated with increased LOS and inflation-adjusted hospital costs, whereas hospitalization in medium and large size hospitals were associated with shortened LOS and decreased inflation-adjusted hospital cost. Both LOS and inflation-adjusted hospital cost did not change significantly with each calendar year increase during the study period.

 

**Table 5:** Predictors of length of stay and inflation adjusted hospital cost among pediatric hospitalizations with intussusception requiring surgery

Variables	Coefficient	Standard Error	95% Confidence Interval		P	Coefficient	Standard Error	95% Confidence Interval		P
			Lower limit	Upper limit				Lower limit	Upper limit	
Year	-0.1	0.1	-0.3	0.04	0.1	259	243	-216	734	0.3
Age										
<1 year	Reference					Reference				
1- 2 year	0.2	0.4	-0.7	1.09	0.6	-661	1203	-3020	1698	0.6
2-4 year	-0.2	0.4	-1.0	0.64	0.7	-242	1121	-2439	1954	0.8
>4 year	1.1	0.4	0.3	1.95	0.01	2852	1122	653	5052	0.01
Gender										
Male	Reference					Reference				
Female	-0.003	0.3	-0.6	0.6	1.0	-303	853	-1975	1369	0.7
Severe disease	4.0	0.5	3.0	4.9	<.0001	15067	1341	12439	17695	<.0001
Gastrointestinal complications	0.5	0.4	-0.3	1.4	0.2	-105	1172	-2403	2192	0.9
Chronic medical conditions childhood	8.8	0.7	7.3	10.2	<.0001	21015	1978	17138	24892	<.0001
Complications from surgery	4.3	0.5	3.3	5.3	<.0001	8511	1453	5664	11358	<.0001
Transfer from outside facility (Acute care hospital/Facility)										
No	Reference					Reference				
Yes	1.2	0.4	0.4	2.0	0.004	4776	1120	2580	6972	<.0001
Primary payer										
Private	Reference					Reference				
Medicaid/Self-pay/Others	-0.1	0.3	-0.7	0.5	0.8	-748	841	-2397	902	0.4
Hospital Region										
Northeast	Reference					Reference				
Midwest	0.8	0.6	-0.3	1.9	0.1	256	1575	-2830	3342	0.9
South	-0.1	0.4	-0.9	0.7	0.8	-1350	1228	-3757	1057	0.3
West	-0.04	0.5	-0.9	0.9	0.9	3030	1352	380	5681	0.03
Hospital bed size								0	0	
Small	Reference					Reference				
Medium	-1.3	0.6	-2.5	-0.1	0.04	-4259	1781	-7749	-769	0.02
Large	-1.3	0.5	-2.3	-0.2	0.02	-6492	1548	-9526	-3458	<.0001
Hospital location & Teaching status										
Non-Teaching (Urban and rural)	Reference					Reference				
Urban - Teaching	0.6	0.4	-0.2	1.4	0.1	1680	1100	-475	3835	0.1

## Discussion

 

A recent meta-analysis demonstrated that outpatient management of pediatric intussusception is safe [14]. The impact of this growing trend on surgical intervention rates for pediatric intussusception is unknown in the US. In the current study, we utilized a nationally representative database to study the trends of pediatric intussusception hospitalizations requiring surgical intervention and the associated resource utilization in the US from 2005-2014. We demonstrated that surgical management rates were highest in males, infants, and Hispanic race/ethnicity. Age ≥1 year and admission in large and urban teaching hospitals were associated with decreased odds of surgical intervention. These findings concur with what is already known about surgical management of intussusception in children [10,15]. However, the present study extends our knowledge on the management of intussusception by examining the trends in surgical intervention and resource utilization in addition to examining the patient and hospital level factors associated with surgical intervention. We found no statistically significant trend in the overall rate of surgical intervention for pediatric hospitalizations with intussusception from 2005-2014. However, there was a significant decline in the rate of surgical intervention for infants (age <1 year) with intussusception. The surgical management of pediatric intussusception remains resource-intensive and there was no significant change in LOS and hospital cost.

Since the first description of the non-operative reduction of intussusception by Harald Hirschsprung in 1905, enema reduction using air or liquid for uncomplicated cases has increased dramatically with a reported success rate of 52-95%, depending on the method used and the population studied [16-18]. Contrary to our hypothesis, we found no significant change in surgical intervention rates for pediatric intussusception from 2005-2014. The reason for this finding is not readily apparent and neither could we find a reason from the published literature to explain this. For the overall rate of surgery to decline significantly, the proportion managed with primary surgery would be expected to decrease from 2005-2014. However, we found that 59% of all the intussusceptions hospitalizations with a procedure code were managed with primary surgery (without a preceding non-operative reduction code) although only 11.1% had severe disease for which surgery is indicated. This proportion of 59% treated with primary surgery is similar to the rate of 58% previously reported for the US from 2000-2003 by Jen et al [15]. Notwithstanding this, there was a significant decline in the surgical intervention rate for infants, the age group with the highest incidence of intussusception. In a previous population-based study with data from 16 states in the US, Tate and colleagues found that the overall rate of surgically treated intussusception in infants declined by 38% from 1993-2004 [3]. This is explained by the observation that the proportion of infants with intussusception undergoing radiologic procedure is increasing and the proportion of intussusceptions due to a pathological lesion at the lead point increases with age, from 5% in infants to 60% in the 5-14 year age group [5,19]. There is an identifiable lesion (pathological lead points) in the majority of children over 5 years of age with intussusception which is less likely to be reduced by enema [19]. This is consistent with the current study finding that age >4 years was associated with increased odds of surgery.

In the present study, 38.5% of intussusception hospitalizations were Whites while 23% were Hispanics. However, the rate of surgery in Hispanics was almost twice that of Whites. Does this suggest that Hispanics tend to develop severe disease? Further studies are needed to explore the relationship between race and surgery for intussusception. One plausible explanation for this observation may stem from racial disparities in healthcare in the US. Hispanic children are more likely to be uninsured than African-American and non-Hispanic Whites [20]. Uninsured children are less likely to receive routine healthcare visits and therefore are less likely to be seeking care when needed [21]. Thus, Hispanic children with intussusception have a disproportionately higher rate of surgery due to limited access to care, leading to delay in seeking care - a proven risk factor for surgery in pediatric intussusception [22].

Previous studies have examined the association between clinical and radiological variables and the odds of surgery in intussusception patients [23]. However, only a few have studied the impact of patient demographic and hospital-level factors on the likelihood of surgery [10,22,24]. The present study is the largest population-based study to examine the patient and hospital-level factors associated with the odds of surgical intervention for pediatric intussusception. We found that age >4 years and severe disease were associated with increased odds of surgery which is consistent with previous studies [19,25]. When compared to the Northeast, intussusception hospitalizations in each of the other census regions were associated with increased odds of surgery. Children’s hospitals are associated with decreased odds for surgical intervention of intussusception than general hospitals [20]. However, the majority of children in the US are hospitalized in general hospitals and the Northeast has the least number of general hospitals [26]. This may account for the increased odds of surgery in other census regions than the Northeast. Moreover, hospitalization at large and urban teaching hospitals was associated with decreased odds of having surgical intervention. This is not surprising because most of the free-standing children’s hospitals in the US are located in large, metropolitan areas and the majority of pediatric radiologists who perform enema reduction are in large cities and academic centers [26-27].

There was no significant change in the median LOS and the inflation-adjusted hospital cost for surgical management of intussusception. This is not surprising because LOS is directly related to hospital cost. This is disappointing given concerns about the rising healthcare costs in the US [28]. Non-operative reduction costs less than surgery and the cost for surgical management of intussusception can be reduced by significantly reducing the proportion managed with primary surgery. This includes but is not limited to improving access to healthcare for children especially minorities, resourcing general hospitals with the capability to perform enema reductions and prompt transfer of patients from facilities that lack the resources to manage intussusception.

The limitations of studies performed with administrative data are well described [29]. A subset of patients with intussusception are treated and discharged from the ER after a short period of observation. These patients are not hospitalized and thus not captured in the NIS database. If they were, the proportion of intussusception hospitalizations managed with surgery would be far lower than we found. However, we used rates per 100,000 population to represent the surgical intervention rate, which is unaffected by the number or the proportion that was discharged from the ER. Intussusception is one of several differential diagnoses for abdominal pain and/or rectal bleeding in children. It is possible that some hospitalizations had the ICD-9-CM code for intussusception, but this was ultimately ruled out. To exclude these, hospitalizations with intussusception had to have a procedure code for enema/pneumatic reduction and/or surgery. Furthermore, we could not determine the impact of potential confounders such as hospital volume for the procedure, type of hospital (children’s vs. non-children's hospital), and duration of symptoms on the odds of surgical intervention. Moreover, we could not ascertain the surgical approach (laparotomy vs laparoscopy) and its impact on LOS and hospital cost. Although both approaches are safe and effective in treating intussusception, laparoscopy was associated with shorter LOS in a previous single center study [30]. Notwithstanding these limitations, the present study is the largest to trend the surgery and resource utilization rates for pediatric intussusception in the US using a nationally representative healthcare database. Further, we evaluated the effect of various patient and hospital-level factors on the odds of surgery.

## Conclusions

Using a nationally representative database, we demonstrated that the overall surgical intervention rate for pediatric intussusception remained unchanged in the US from 2005-2014. However, the rate significantly declined in infants, the age group with the highest incidence of intussusception. Furthermore, LOS and inflation-adjusted hospital cost remained stable without any trends. The proportion of intussusception hospitalizations managed with primary surgery remains high and further studies are needed to identify the possible factors. An understanding of the indications could potentially help lower the overall surgical intervention rate in addition to reducing resource utilization for intussusception in the US.

## References

[1] Jiang J, Jiang B, Parashar U, Nguyen T, Bines J, Patel MM (2013). Childhood intussusception: A literature review. PLoS One.

[2] Beasley SW (2017). The ‘ins’ and ‘outs’ of intussusception: Where best practice reduces the need for surgery. J Paediatr Child Health.

[3] Tate JE, Simonsen L, Viboud C (2008). Trends in intussusception hospitalizations among US infants, 1993-2004: Implications for monitoring the safety of the new rotavirus vaccination program. Pediatrics.

[4] Gilmore AW, Reed M, Tenenbein M (2011). Management of childhood intussusception after reduction by enema. Am J Emerg Med.

[5] Zickafoose JS, Benneyworth BD, Riebschleger MP, Espinosa CM, Davis MM (2012). Hospitalizations for intussusception before and after the reintroduction of rotavirus vaccine in the United States. Arch Pediatr Adolesc Med.

[6] INTRODUCTION TO THE HCUP NATIONAL INPATIENT SAMPLE (NIS) 2014.2016. http://www.hcup-us.ahrq.gov (2020). Introduction to The Healthcare Cost and Utilization Project (HCUP) National Inpatient Sample and Agency for Healthcare Research and Quality. http://www.hcup-us.ahrq.gov.

[7] Bhatt P, Bray L, Raju S (2019). Temporal trends of pediatric hospitalizations with acute disseminated encephalomyelitis in the United States: An analysis from 2006 to 2014 using national inpatient sample. J Pediatr.

[8] Tate JE, Yen C, Steiner CA, Cortese MM, Parashar UD (2016). Intussusception rates before and after the introduction of rotavirus vaccine. Pediatrics.

[9] Schollin Ask L, Svensson JF, Olén O, Örtqvist Å (2019). Clinical presentation of intussusception in Swedish children under 3 years of age and the validity of diagnostic coding. Pediatr Surg Int.

[10] McAteer JP, Kwon S, Lariviere CA, Oldham KT, Goldin AB (2013). Pediatric apecialist care is associated with a lower risk of bowel resection in children with intussusception: A population-based analysis. J Am Coll Surg.

[11] Feudtner C, Hays RM, Haynes G, Geyer JR, Neff JM, Koepsell TD (2001). Deaths attributed to pediatric complex chronic conditions: national trends and implications for supportive care services. Pediatrics.

[12] (2020). HCUP-US Cost-to-Charge Ratio Files. http://www.hcup-us.ahrq.gov/db/state/costtocharge.jsp.

[13] (2019). CDC WONDER. http://wonder.cdc.gov.

[14] Litz CN, Amankwah EK, Polo RL, Sakmar KA, Danielson PD, Chandler NM (2019). Outpatient management of intussusception: a systematic review and meta-analysis. J Pediatr Surg.

[15] Jen HC, Shew SB (2009). The impact of hospital type and experience on the operative utilization in pediatric intussusception: a nationwide study. J Pediatr Surg.

[16] Davis CF, McCabe AJ, Raine PAM (2003;38 (7 Suppl)). The ins and outs of intussusception: History and management over the past fifty years. J Pediatr Surg.

[17] Stein-Wexler R, O’Connor R, Daldrup-Link H, Wootton-Gorges SL (2015). Current methods for reducing intussusception: survey results. Pediatr Radiol.

[18] Bekdash B, Marven SS, Sprigg A (2013). Reduction of intussusception: Defining a better index of successful non-operative treatment. Pediatr Radiol.

[19] Blakelock RT, Beasley SW (1998). The clinical implications of non-idiopathic intussusception. Pediatr Surg Int.

[20] Zambrana RE, Logie LA (2000). Latino child health: Need for inclusion in the US national discourse. Am J Public Health.

[21] Margaret Edmunds (2020). America's children: Health insurance and access to care. National Research Council (US) and Institute of Medicine (US); Committee on Children, Health Insurance, and Access to Care.

[22] Blackwood BP, Theodorou CM, Hebal F, Hunter M CJ (2016). Pediatric intussusception: Decreased surgical risk with timely transfer to a children’s hospital. J Pediatr Care.

[23] Fallon SC, Lopez ME, Zhang W (2013). Risk factors for surgery in pediatric intussusception in the era of pneumatic reduction. J Pediatr Surg.

[24] Somme S, To T, Langer JC (2006). Factors determining the need for operative reduction in children with intussusception: a population-based study. J Pediatr Surg.

[25] Savoie KB, Thomas F, Nouer SS, Langham MR, Huang EY (2017). Age at presentation and management of pediatric intussusception: A pediatric health information system database study. Surg (United States).

[26] Leyenaar JAK, Ralston SL, Shieh MS, Pekow PS, Mangione-Smith R, Lindenauer PK (2016). Epidemiology of pediatric hospitalizations at general hospitals and freestanding children’s hospitals in the United States. J Hosp Med.

[27] Merewitz L, Sunshine JH (2006). A portrait of pediatric radiologists in the United States. Am J Roentgenol.

[28] Moses H, Matheson DHM, Dorsey ER, George BP, Sadoff D, Yoshimura S (2013). The anatomy of health care in the United States. JAMA - J Am Med Assoc.

[29] Gavrielov-Yusim N, Friger M (2014). Use of administrative medical databases in population-based research. J Epidemiol Community Health.

[30] Bailey KA, Wales PW, Gerstle JT (2007). Laparoscopic versus open reduction of intussusception in children: a single-institution comparative experience. J Pediatr Surg.

